# Aromatase Deficiency in Two Siblings with 46,XX Karyotype Raised as Different Genders: A Novel Mutation (p.R115X) in the *CYP19A1* Gene

**DOI:** 10.4274/jcrpe.galenos.2019.2018.0198

**Published:** 2020-03-19

**Authors:** Samim Özen, Tahir Atik, Özlem Korkmaz, Hüseyin Onay, Damla Gökşen, Ferda Özkınay, Özgür Çoğulu, Şükran Darcan

**Affiliations:** 1 Ege University Faculty of Medicine, Department of Pediatric Endocrinology, İzmir, Turkey; 2 Ege University Faculty of Medicine, Department of Pediatrics, Division of Pediatric Genetics, İzmir, Turkey; 3 Ege University Faculty of Medicine, Department of Medical Genetics, İzmir, Turkey

**Keywords:** 46, XX disorder of sex development, aromatase deficiency, CYP19A1 gene

## Abstract

Aromatase deficiency rarely causes a 46,XX sexual differentiation disorder. The *CYP19A1* gene encodes the aromatase enzyme which catalyses the conversion of androgens to oestrogens. In cases with 46,XX karyotype, mutations in the *CYP19A1* gene can lead to disorders of sex development. Clinical findings in aromatase deficiency vary depending on the degree of deficiency. The effect of increased androgens, including acne, cliteromegaly and hirsutism, can be observed in mothers with placental aromatase deficiency. A decrease in maternal virilisation symptoms is observable in the postpartum period. It is rarely reported that there is no virilization in pregnancy. In this study, two 46,XX sibling having the p.R115X (c.343 C>T) novel pathogenic variant in the *CYP19A1* gene and raised as different genders, with no maternal virilisation during pregnancy, are presented. In conclusion, 46,XX virilised females should be examined in terms of aromatase deficiency once congenital adrenal hyperplasia has been excluded, even if there is no history of maternal virilisation during pregnancy.

**What is already known on this topic?**

The *CYP19A1* gene encodes the aromatase enzyme which catalyses the conversion of androgens to oestrogens. In cases with 46,XX karyotype, mutations in the *CYP19A1* gene can lead to disorders of sex development.

**What this study adds?**

Two 46,XX sibling having the novel p.R115X (c.343 C>T) pathogenic variant in the *CYP19A1* gene and raised as different genders are presented. This variant was not associated with maternal gestational virilisation.

## Introduction

Aromatase deficiency is a rare autosomal recessive disorder caused by mutations in the *CYP19A1* gene (1). The *CYP19A1* gene encodes the aromatase enzyme which catalyses the conversion of androgens to oestrogens. In the affected 46,XX cases, clinical findings in the neonatal period vary between mild cliteromegaly and complete labioscrotal fusion due to differences in exposure to increased androgens in the intrauterine phase. An increase in virilisation at puberty or the non-appearance of secondary sex characteristics, are the main clinical features in the late period. Affected 46,XY cases have normal prepubertal growth. Delayed epiphyseal closure, eunuchoid body structure and a decrease in bone mineral density can be observed in both sexes (2). This study presents a novel pathogenic variant in the *CYP19A1* gene in two 46,XX siblings raised as different genders.

## Case Reports

### Case 1

**Figure 1:**
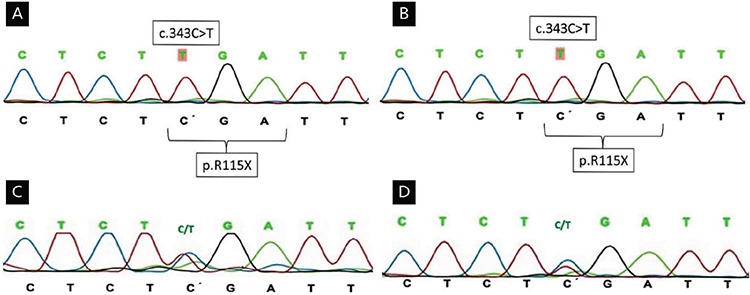
**A) (Case 1), B) (Case 2):** A novel homozygous nonsense pathogenic variant p.R115X (c.343 C>T) was detected in the *CYP19A1* gene sequence analysis. **C)** (Mother), **D)** (Father): The parents were heterozygous for the same mutation

A 14-year-old patient who had been raised as a male was brought to the pediatric endocrinology clinic for undescended testis and hypospadias. Although parental consanguinity was not reported to be present, the family history revealed that they were living in a village of only 500 inhabitants. The patient’s mother, who was first pregnancy and primigravida, had no symptoms during pregnancy of excessive androgen production, such as hair loss, virilisation, or acne. On physical examination, height, weight, and phallus were measured to be 154.9 cm [standard deviation score (SDS): -2.5], 57 kg (SDS: -0.6), and 2 cm respectively. Breast tissue and palpable gonads were not detected. Prader stage 3, two urogenital openings and stage 2 pubic pilosity were also noted. On laboratory examination, bone age was 11 years. Gonadotropin concentrations were: follicle stimulating hormone (FSH) 70 mIU/mL (1.5-12.8 mIU/L); luteinizing hormone (LH) 30 mIU/mL (0.1-12 mIU/mL); free testosterone 0.9 pg/mL (0.8-1.4 pg/mL); and estradiol 22.9 pg/mL (7-60 pg/mL). Adrenocorticotropic hormone (ACTH), cortisol and 17-hydroxyprogesterone (17-OHP) were all found to be in the normal range. Pelvic ultrasonography (USG) revealed 19x14 mm right ovary and 15x12 mm left ovary and an absence of uterus. Karyotype was 46,XX and no variants were found in the *SRY* gene on fluorescence in situ hybridization (FISH) analysis. On laparoscopic examination normal-looking bilateral ovaries and a small uterus were observed. The biopsy findings of the right gonad were consistent with ovarian tissue and ovarian follicle cysts were observed. Sequence analysis of the *SOX9* gene revealed no mutation. Clinical and laboratory findings of the patient suggested aromatase deficiency and a novel homozygous nonsense p.R115X (c.343 C>T) pathogenic variant was found on Sanger sequencing of the *CYP19A1* gene (Figure 1A). Both parents were heterozygous for the same mutation (Figure 1C, 1D). There were no unusual clinical findings in the parents. The mutation found in the cases was predicted to be pathogenic by *in* *silico* analysis by Varsome program (https://varsome.com/).

The Institutional Council of Disorders of Sex Development decided that the case should be raised male on the ground of a more distinct male sexual identity. Salpingo-oophorectomy, hysterectomy and genitoplasty were performed. Intramuscular testosterone propionate and testosterone phenylpropionate treatments were administered with a 100 mg/month starting dose and gradually increased every six months. Oral estradiol hemihydrate treatment of 0.25 mg/day was initiated in the follow-up. At the age of 21, bilateral testicular prostheses were surgically implanted. During follow-up bone mineral densitometry showed early onset osteoporosis (L1-L4 bone mineral density Z score: -2,2) and oral calcium supplementation was given. Calcium, phosphorus, parathyroid hormone (PTH) and vitamin D concentrations were within normal limit. At the age of 22, weight, height, and phallus were measured to be 86.6 kg (SDS: 1.29), 173.5 cm (SDS: -0.43) and 7 cm respectively.

### Case 2

The eight year-old sibling of Case 1, who had been raised as a female. On physical examination, height and weight were 125.5 cm (SDS: -0.3), 22.3 kg (SDS: -0.9) respectively. Phallus was measured to be 1 cm. There were no palpable gonads, two urogenital openings and stage 1 pubic pilosity were also noted. Pubertal development was found to be stage 2 according to Prader score. Similar to the other sibling, the bone age of Case 2 was found to be retarded (5 years 9 months). FSH concentration was 22 mIU/mL (1.0-4.2 mIU/mL), LH: 30 mIU/mL (0.1-0.3 mIU/mL), free testosterone 0.2 pg/mL (0.15-0.6 pg/mL), estradiol: 5 pg/mL (N<15). The patient had normal ACTH, cortisol and 17-OHP. Uterus and left ovary were not visualized on pelvic USG whereas a 12-mm right ovary was identified. Karyotype was found to be 46,XX. FISH analysis showed no variants in the *SRY* gene. The same homozygous pathogenic variant in the *CYP19A1* gene was also detected in this sibling (Figure 1B). The Institutional Council of Disorders of Sex Development recommended the case to be raised as a female, on the grounds that female sexual identity was more distinct. L1-L4 Z score was found to be -2.4 on bone mineral densitometry during the follow-up period. Calcium, phosphorus, PTH and vitamin D concentrations were within normal limits but oral intake of calcium was increased. At the age of 11, oral estradiol hemihydrate treatment was begun with 0.25 mg/day starting dose and was gradually increased every six months. At the age of 16, physical examination showed a weight of 58.7 kg (SDS: 0.28), height 160 cm (SDS: -0.44) and stage 5 puberty. Routine pelvic USG showed a uterus with the dimensions of 62x35 mm. The patient was treated with a combination of oestrogen and progesterone. After this treatment she had menarche and regular menstural cycles.

The parents of the patients were informed about the diagnosis and consent for laboratory analyses and publication were obtained.

## Discussion

Aromatase is a member of the cytochrome P450 superfamily that catalyses a hydroxylation reaction in which an oxygen atom is attached to an organic molecule (3). The human aromatase enzyme (P450C19) is the product of *CYP19A1* gene that converts androgens (P19) to oestrogens (P18) and is a microsomal enzyme responsible for oestrogen synthesis in all vertebrates (3,4). The enzyme-encoding gene is composed of 10 exons (5). Mutations in the *CYP19A1* gene lead to loss of enzyme function and decrease in oestrogen synthesis. Most of the reported mutations contain single base changes in exons (6,7). In the study, the *CYP19A1* gene sequence analysis detected homozygous novel nonsense p.R115X pathogenic variant in both siblings (Figure 1A, 1B). This nonsense mutation is predicted to be pathogenic using *in silico* analysis (MutationTaster) (8) and minor allele frequency data in several public databases including the NCBI dbSNPbuild141 (http://www.ncbi.nlm.nih.gov/SNP/), the 1000 Genomes Project (http://www.1000genomes.org/) and the Exome Aggregation Consortium (http://exac.broadinstitute.org/).

Clinical findings in aromatase deficiency vary depending on the retained proportion of enzyme function. Due to the effect of increased androgens caused by placental aromatase deficiency, acne, cliteromegaly and hirsutism can be observed in mothers carrying affected fetuses. A decrease in the symptoms of maternal virilisation is observed in the postpartum period (9). Placental aromatase activity of as little as 1-2% is reported to be protective against maternal virilisation during pregnancy (10). In the family presented here, the mother had no symptoms of excessive androgen production during pregnancy. Enzyme activity was not studied in the patients nor their mother. Marino et al (11) reported that maternal virilisation was also absent in their three cases with *CYP19A1 *mutations. During the follow-up period, phenotypic variability was determined among the affected patients. Two patients had a new mutation (c.574C>T). They found c.628G>A mutation in four of the six unrelated patients.

It has been reported that of 24 patients (12 males, 12 females) with proven *CYP19A1* deficiency, 70% of the females showed virilisation compatible with Prader stage 4-5, while males usually presented with metabolic problems and short stature (7,12). For affected female cases, variable phenotype, such as cliteromegaly due to increased androgen levels in the intrauterine phase or complete labioscrotal fusion can be observed. It has been suggested that in aromatase-deficient prepubertal girls, an amplification of FSH signalling might occur in the presence of high intra-ovarian androgen production and be responsible for the development of ovarian follicular cysts (3). On the other hand, hypoplastic ovaries rather than enlarged ovaries in aromatase-deficient females have rarely been reported. Lin et al (13) and Akçurin et al (14) reported a few cases of aromatase deficiency with hypoplastic ovaries and uterus. Lin et al (13) suggested that the streak ovaries may be an inherent manifestation of *CYP19A1* deficiency. Also, polycystic ovaries may appear in later periods depending on human chorionic gonadotropin stimulation. Cliteromegaly, hirsutism and acne can be seen in affected individuals with the non-appearance of secondary sex characteristics in the adolescence period (3). The studies have showed that loss of function mutations in the gene may result in various phenotypic changes, especially appearing in the pre-pubertal and pubertal period (11). In the study of Lin et al (13) it was demonstrated that human aromatase mutations may produce variable or “non-classic” phenotypes. They reported that low residual aromatase activity may be sufficient for the development of breast and uterus in adolescence, despite significant androgenization in the uterus. Such phenotypic variability can be further influenced by modifying factors such as non-classical pathways of estrogen synthesis, variability in the core modifiers, or differences in androgen responses. The siblings presented in this study had been raised as different genders due to the appearance of their external genitalia and virilisation levels.

In aromatase deficiency, oestrogen replacement treatment regulates gonadotropin secretion, glucose metabolism and liver functions while reducing lipid and insulin levels (14,15). In our cases, lipid levels and glucose metabolism were found to be normal. However, decreased FSH and LH levels were observed with the oestrogen replacement treatment. Bone mineralisation and maturation are adversely affected in patients with aromatase deficiency. Oestrogen has positive effects on bone density by prolonging the life cycles of osteoblasts and osteocytes while reducing bone resorption (4). Osteoporosis was detected in both of our patients. Hormone replacement therapy was initiated and oral intake of calcium was increased as they were followed up.

In conclusion; 46,XX virilised cases should be examined in terms of aromatase deficiency after congenital adrenal hyperplasia has been excluded, even if there was no maternal history of virilisation during pregnancy. This should include *CYP19A1* mutation analysis. Early diagnosis of this disorder is of vital importance for gender selection and hormone replacement therapy.
